# Chronic Cholecystitis: Its Cause, Complications and Sequelae1Read at the forty-first annual meeting of the Medical Association of Central New York, held at Buffalo. October 27, 1908.

**Published:** 1909-08

**Authors:** H. M. Spofford

**Affiliations:** Batavia, N. Y.


### Chronic Cholecystitis: Its Cause, Complications and Sequelae[1]

BY H. M, SPOFFORD, Batavia, N. Y.

BEFORE I am through you may think I have digressed sadly from the subject indicated by the title. However the reason that I think the title appropriate is that the clinical picture the physician has before him not only of the case cited below, but the average one named by the subject is that of chronic cholecystitis with its accompanying jaundice. Having had within the past year occasion to follow throughout a case of chronic cholecystitis and to look up some of the recent literature on the subject, the question arises what might be the primary causative factor of the condition. It is my purpose to show if possible by a short history of what would ordinarily be called a typical case of chronic jaundice, and citing a few similar instances that the cholecystitis is not necessarily a primary condition *per se,* but is due in many cases to inflammation and swelling of the head of the pancreas, which is seldom considered as the prime causative factor not only in cases of chronic and subacute but in acute cholecystitis.

CASE.—Male, age 33; weight, 130 lbs.; mother died of phthisis thirty years previously. No venereal disease; no alcoholism. About November 20, 1907, he began to have headaches and spells of nausea, gradually increasing in frequency through a period of two or three weeks until they occurred daily. He had continued with his work, however, until his condition became so troublesome that advice was solicited. The attending physician found temperature 102½°, pulse, slow; severe headache, constipation; and typhoid was suspected. The condition remained unchanged for three or four days, when jaundice appeared with the usual symptoms of vomiting, obstipation, pulse 40, loss of appetite and gen-

---

1. Read at the forty-first annual meeting of the Medical Association of Central New York, held at Buffalo. October 27, 1908.

eral discomfort. The usual treatment had no effect. The jaundice deepened, pruritis and insomnia became very marked, urine almost black, and the stools were bulky, greasy and clay colored. At the end of a week or ten days more the nausea disappeared, and in its place was the most intense craving for food which was not genuine hunger, and was not satisfied by the ingestion of food. The weight dropped from 130 to 112. Physical examination revealed a dark bronze-like icterus over the entire body surface, liver dulness one finger breadth below the costal margin, tender over the gall-bladder, which could apparently be palpated. In the median line, two inches above the umbilicus was a small area especially tender to the touch, and deep palpation as could be borne gave an indefinite sense of resistance to the examiner as of a deeply located hard mass.

About the middle of December the patient was taken to Dr. Allen A. Jones, of Buffalo, for his opinion. The case was again diagnosticated as subacute cholecystitis with prognosis good for probable recovery within a few weeks. The condition, however, remained unchanged except for the jaundice becoming deeper until January 13, 1908, when operation was decided upon, and upon January 20, performed by Dr. William J. Mayo, of Rochester, Minn. On opening the abdomen the liver was found enlarged and soft, probably a beginning hypertrophic cirrhosis, spleen large and soft, gall-bladder normal in size and healthy looking, common duct small and walls apparently thickened. The head of the pancreas surrounding the common duct was enlarged, showing a condition of catarrhal pancreatitis. On opening the gall-bladder was found thick black ropy bile, some particles of probable stone-forming material scattered throughout, and a few yellow areas of beginning degeneration in the mucous membrane. The cystic and hepatic ducts were patent, and the common duct closed by pressure from the swollen head of the pancreas. No stones. The gall-bladder was drained, the tube being removed on the tenth day, patient up on the eleventh day and left hospital on the twelfth. The drainage continued for about a week longer, the wound then closed entirely. The jaundice quickly disappeared, the patient gaining about a pound daily until he had regained his former weight, and up to this time is in good health.

Now it is my intention to try and emphasize, that in severe cases of catarrhal jaundice of which I think I have given a good type that do not clear up, may be in many cases, due to pancreatic swelling thereby obstructing the common duct and not to a true colangitis from gallstones or other excitant within the duct itself; and in so doing I must of necessity borrow somewhat from the literature of far greater experience than my own.

To substantiate, I found that in nearly two-thirds of the cases operated for cholelithiasis and accompanying jaundice by Mayo Robson the presence of pancreatitis was practically proven before operation, by the chemical and microscopical examination of the feces and urine.

Further, that the common duct passed not behind but was surrounded by the head of the pancreas in about two-thirds of the cases anatomically reported by Helly, and according to the above mentioned operator's observation this relation held practically parallel in the reports of about 200 cases. Again, that we have chronic jaundice in cases where the stones are in the gall-bladder and cystic duct with typical reoccurring attacks of biliary colic, the common duct being patent except for pressure from a hard and swollen pancreas surrounding the lower portion.

Further, that in cases of cholelithiasis with jaundice the ictures sometimes remains after the removal of the stones. A case cited in a condition of this sort was subsequently operated upon, a cholecystenterostomy performed, the jaundice clearing up with complete recovery. Again that pancreatic swelling demonstrated by operation has kept up a jaundice after the stones had passed into the duodenum and been found in the stools during and following an attack of colic. Lastly, that in the case described by me the condition was accurately diagnosticated by Dr. William J. Mayo before operation, not as a cholecystitis or cholangitis, but as a pancreatitis as the primary condition.

Now, if what I infer is correct, I wish to say a word as to the early diagnosis of what I presume to say in many cases is the primary condition. The diagnosis of catarrhal pancreatitis or chronic interstitial pancreatitis has always been considered difficult and I presume to say that one of the chief difficulties to many of us has been that picture of cholecystitis and jaundice blinding us from looking farther ahead than the icteric skin and putty stool. A careful chemical examination of the feces will always reveal neutral fat in pancreatic pathology, the type of which I have mentioned. Even should we not go so far as a chemical examination, the presence of a bulky greasy stool should cause us to suspect pancreatic change. (It is bound to be clay colored in jaundice). With continued jaundice loss of weight, liver enlargement, gastro-intestinal disturbances and lack of pain to assist us in the diagnosis, to me one of the prime diagnostic points is a small exquisitely sensative area in the median line about two inches above the umbilicus which even on moderate palpation gives a feeling of indefinite localised resistance to the examining finger.

Finally, the Cambridge test of the urine in which has demonstrated prior to operation that pancreatic disease existed in most of the cases of Mayo Robson since 1900. To give this process of obtaining the so-called pancreatic reaction, or of demonstrating the presence of pancreatic crystals, would take so long that I will not read it. Suffice it to say that it could be worked out in any well equipped chemical laboratory. (Surgery and Pathology of the Pancreas recently published by Saunders gives the laboratory technic.)

A word as to treatment: practically, every case of chronic jaundice operated upon in which pancreatic swelling was demonstrated has been cured by drainage of the gall-bladder indirectly relieving the primary trouble. The object is drainage and drainage sufficiently long to insure the return to normal of the pancreas, gall-bladder, bile ducts, liver, or any structure so histologically or anatomically related as to have been influenced by the condition. My only regret in the treatment of the case cited, if there be any, is that a cholecystenterostomy was not performed to insure perfect drainage throughout the future. I believe surgery has been the means of demonstrating more the true pathology of chronic cholecystitis within the last fifteen years than all the post mortem pathology taught us before, since, and to surgery I think we shall look in the future for the cure of that condition which we may hope to diagnosticate in its earlier stages. We shall then see that pathology in the beginning and not as we have at post mortem when the complications and sequelæ have become so prominent that it is impossible to tell what the primary pathology really was.

